# Ultralight graphene oxide/polyvinyl alcohol aerogel for broadband and tuneable acoustic properties

**DOI:** 10.1038/s41598-021-90101-0

**Published:** 2021-05-19

**Authors:** Mario Rapisarda, Gian-Piero Malfense Fierro, Michele Meo

**Affiliations:** Department of Mechanical Engineering, University of Bath, Bath, BA27AY UK

**Keywords:** Acoustics, Graphene, Gels and hydrogels, Composites

## Abstract

An ultralight graphene oxide (GO)/polyvinyl alcohol (PVA) aerogel (GPA) is proposed as a new class of acoustic materials with tuneable and broadband sound absorption and sound transmission losses. The interaction between GO sheets and PVA molecules is exploited in our environmentally friendly manufacturing process to fabricate aerogels with hierarchical and tuneable porosity embedded in a honeycomb scaffold. The aerogels possess an enhanced ability to dissipate sound energy, with an extremely low density of 2.10 kg m^−3^, one of the lowest values ever reported for acoustic materials. We have first experimentally evaluated and optimised the effects of composition and thickness on the acoustic properties, namely sound absorption and sound transmission losses. Subsequently, we have employed a semi-analytical approach to evaluate the effect of different processing times on acoustic properties and assessed the relationships between the acoustic and non-acoustic properties of the materials. Over the 400–2500 Hz range, the reported average sound absorption coefficients are as high as 0.79, while the average sound transmission losses can reach 15.8 dB. We envisage that our subwavelength thin and light aerogel-based materials will possess other functional properties such as fire resistance and EMI shielding, and will prove to be novel acoustic materials for advanced engineering applications.

## Introduction

The development of innovative acoustic materials has been of huge interest in the past decades, in particular porous absorbers have been extensively studied and adopted for several engineering applications^1^. Traditional porous absorbers such as cellular foams^2–4^ and fibrous materials^4–6^ exhibit good sound absorption abilities over a medium frequency range (i.e., 800–2000 Hz), but they are typically bulky and heavyweight at lower frequencies, limiting their application. Porous absorbers dissipate sound energy due to two main mechanisms: viscous friction on pore walls and thermal losses within pores^7,8^. The understanding of sound absorption capabilities of these materials relies on the prediction of their effective density ($\rho_{e}$) and bulk modulus ($K_{e}$), that can be achieved with a semi-phenomenological fluid model developed by Johnson–Champoux–Allard (JCA)^9,10^. The model relates the sound propagation through porous materials to their non-acoustic properties, which are porosity ($\phi$), flow resistivity ($\sigma$), tortuosity ($\alpha_{\infty }$), viscous ($\Lambda$) and thermal ($\Lambda ^{\prime}$) characteristic lengths. Therefore, tailored absorption of porous materials requires accurate measurement of these factors, linked to a precise manufacturing process. Porous absorbers can be distinguished by chemical composition as organic, hybrid, or inorganic^4^, with a recent growing interest in the use of carbon-based materials^11,12^. Graphene oxide (GO) is an ideal candidate for engineering novel absorbers, thanks to its peculiar chemical structure consisting of a two-dimensional (2D) lattice of sp^2^ hybridised carbon atoms with oxygen functionalities^13^. The main advantages of GO are its capability to form stable suspensions in water^14^ and to be templated in various assemblies such as aerogels^15^ with low cost and in environmentally friendly processes. GO has been evaluated for applications including water treatment^16^, energy storage^17^, composite reinforcements^18^, EMI shielding^19^, and thermal insulation with fire-retardancy^20^. Acoustic-related properties have recently been described^8,12,21^. Nine et al.^21^ developed a hybrid foam with GO supported by Melamine where it promoted an increase in air-flow resistivity and tortuosity leading to a sound absorption coefficient of 0.6 over 800 Hz with a sample thickness of 26 mm. Similarly, Oh et al.^12^ fabricated a directionally antagonistic Graphene Polyurethane aerogel with a broadband absorption coefficient over 0.6 above 1000 Hz with a sample thickness of 30 mm. An example of carbon-only foam is found in the work of Lu et al.^8^, where a bubbled GO solution was freeze-cast and thermally reduced. A Bubbled Graphene Monolith was obtained, with a normalised absorption coefficient of 0.9 in the 800–6300 Hz range with a sample thickness of 30 mm and a density of 7.5 kg m^−3^. While these results achieve broadband absorption with thin structures, the pursuit of tuneable, lighter, and higher absorbing materials is still of fundamental and practical importance.

Herein we present a new class of ultralight and subwavelength thin acoustic aerogels with high, broadband, and tuneable sound absorption and sound transmission loss. These are manufactured by ultra-high shear mixing blends of GO and polyvinyl alcohol (PVA) which are then embedded in a honeycomb (HC) core, freeze-cast, and finally freeze-dried. This process allows the incorporation of air bubbles in a templated structure that leads to the ultralight GO/PVA aerogels (GPAs). PVA has many favourable characteristics such as high chemical resistance, good optical and physical properties^22^, low toxicity^23^ and high biodegradability^24^. In addition, its water solubility and cross-linking ability renders it an ideal candidate to form homogeneous solutions with GO. While alterations of blend composition produce aerogels with different physicochemical characteristics, variations in the time of ultra-high shear mixing changes the structural characteristics. Both directly affect the efficiency of sound dissipation through the material and, consequently, shape the JCA model as a powerful tool for the understanding of the acoustic behaviour of GPAs. Digital Microscopy (DM), Scanning Electron Microscopy (SEM), Fourier-Transform Infrared Spectroscopy (FT-IR) and X-Ray Diffractometry (XRD) are used to characterise the physical and chemical properties of the aerogels. The Normal Absorption Coefficient ($\alpha$) and the Normal Incident Sound Transmission Loss ($STL$) are measured to determine the acoustic properties of the proposed material, evaluating and optimising the effects of composition, thickness and processing time. The JCA model is used to predict the physical parameters of the aerogels, identify the effects of the shear mixing process and ultimately provide information to tune the acoustic properties of the absorbers. To the best of our knowledge, no reports have been published discussing the acoustic behaviour of functionalised GO aerogels based on semi-analytical models, or describing their sound transmission losses. The ultralight aerogels manufactured in this work possess high broadband sound absorption, with the optimised GPA potentially being the lightest porous absorber on record at this time.

## Results

### Formation of ultralight GO/PVA aerogels

**Figure 1:**
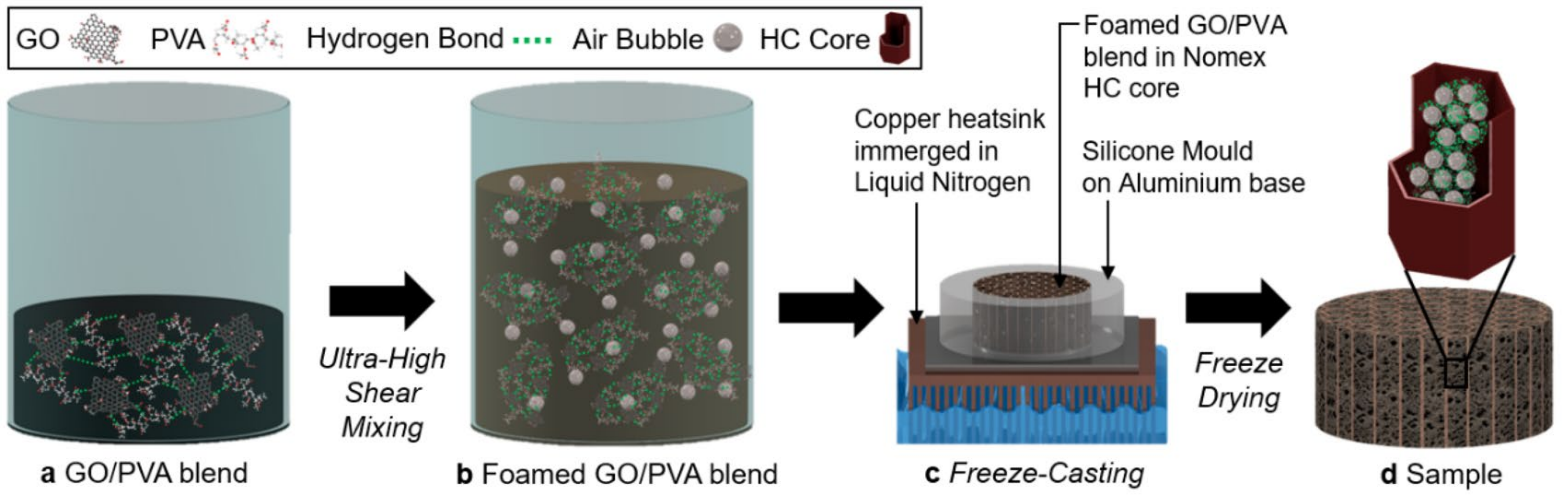
Schematic illustration of the ultralight GPAs.

GO is hydrophilic due to oxygen functionalities such as epoxy, hydroxyl and carboxyl groups which are found on its basal planes and edges^25^. A polar solvent such as water can intercalate between GO interlayer spacings^13,26^, leading to stable suspensions. Likewise, thanks to hydroxyl functionalities, PVA is also hydrophilic and water-soluble^27^. As shown in Fig. 1a, when GO suspensions and PVA solutions are mixed in water, homogeneous blends can be obtained due to hydrogen bonds between the molecules of the two components^28,29^. Figure 1b pictures air bubble entrapment (i.e., foaming) after ultra-high shear mixing of the blends. This is a result of the low interfacial tension of PVA, whereas foam stability is improved by changes to surface elasticity and viscosity due to the presence of GO^30–32^. Increasing the amount of GO increases foaming capability, until a critical concentration of solids leads to bulk clustering of particles that destabilises the foam^33^. After a stable hydrogel is obtained, it is possible to maintain the templated structure and to embed it in a Nomex HC core. Figure 1c,d shows the subsequent freeze-casting and freeze-drying processes: the structure is first frozen unidirectionally from the bottom (i.e., cold surface) to the top (i.e., surface exposed to the atmosphere), resulting in ice crystals growing vertically and pushing the bigger and lighter air bubbles upward; it is then dried though sublimation as pressure and temperature inside the drying chamber are below the triple point. Figure 1d presents the resulting aerogel characterised by a hierarchical porosity. Micro-porosity is generated by the exclusion of particles, polymeric molecules, or a mixture of them, from the nucleation and growth of small ice crystals due to extremely low temperature exposure (i.e., about − 190 °C thanks to the use of Liquid Nitrogen as freezing medium)^34^. Macro-porosity is instead induced by air bubbles previously entrapped.

**Figure 2:**
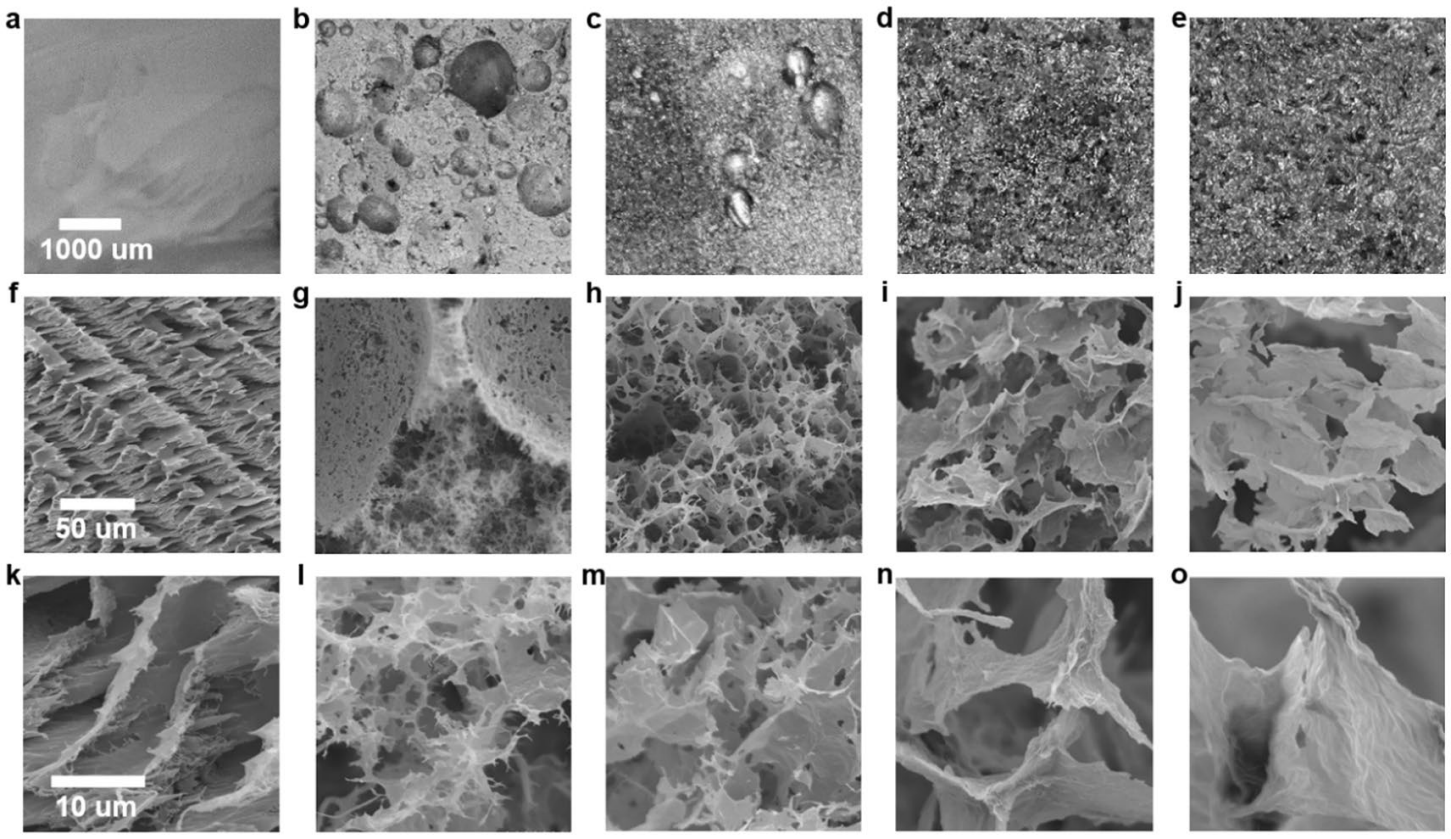
(**a**–**e**) DM and (**f**–**o**) SEM images of GO/PVA aerogels. PVA (**a**,**f**,**k)**, GPA-1 (**b**,**g**,**l**), GPA-2 (**c**,**h**,**m**), GPA-3 (**d**,**i**,**n**), GO (**e**,**j**,**o**). Magnifications: (**a**–**e**) × 20, (**f**–**j**) × 500, and (**k**–**o**) × 3000. Same scale bars apply to images with equal magnification.

In Table 1 GPAs of different composition are compared with pure GO and PVA aerogels. Blends having PVA as the more abundant component have been excluded as the resulting aerogels did not possess acceptable structural robustness. The variation in the amount of PVA in the starting blend leads to both macroscopic (Fig. 2a–e, DM) and microscopic (Fig. 2f–o, SEM) differences in morphology. GPA-1 and GPA-2 possess a similar micro-porous structure, with the first showing the largest macroscopic entrapment of air bubbles. GPA-3 exhibits no bubbles and a bulkier micro-structure, similar to pure GO. The transition from light to bulky structures is reflected by the physical properties of the aerogels, of which GPA-1 is the lightest with a density of 5.11 kg m^−3^ and a porosity of 99.32% (Table 1). A processing time of 15 min is used for the initial blends, as it represents the optimum state of air entrapment, homogenisation and structural robustness in the resulting aerogel. However, the time of ultra-high shear mixing controls air entrapment in the foamed blends, and in so doing, tunes the structural properties of the aerogels to maximise sound energy dissipation. The 1:1 ratio blend shows the best tuning ability in the time interval of 5–20 min, with the resulting physical properties summarised in Table 2. Notably, an ultralight aerogel characterised by a density of 2.10 kg m^−3^ and a porosity of 99.72% is obtained with precisely 5 min of processing. GPAs are among the lightest acoustic materials reported in the literature so far (see Table S1 of Supplementary Information), guaranteeing a small weight increase with respect to the HC core that is as little as the 3.43% for the lightest sample (Tables 1 and 2).

**Table 1:** Physical properties of all samples.

Sample	GO:PVA ratio	Density (kg m^−3^)	${\Phi }$ (%)	Weight increase on HC core (%)
PVA	0:1	43.80	96.5	71.55
GPA-1	1:1	5.11	99.32	8.34
GPA-2	2:1	7.80	98.68	12.74
GPA-3	3:1	7.59	98.71	12.41
GO	1:0	5.80	97.77	11.83

**Table 2:** Physical properties of GPA-1 samples for various processing times.

Processing time (min)	Density (kg m^−3^)	${\Phi }$ (%)	Weight increase on HC core (%)
5	2.10	99.72	3.43
10	4.38	99.42	7.16
15	5.11	99.32	8.34
20	7.41	99.23	12.11

### Physicochemical characterisation

**Figure 3:**
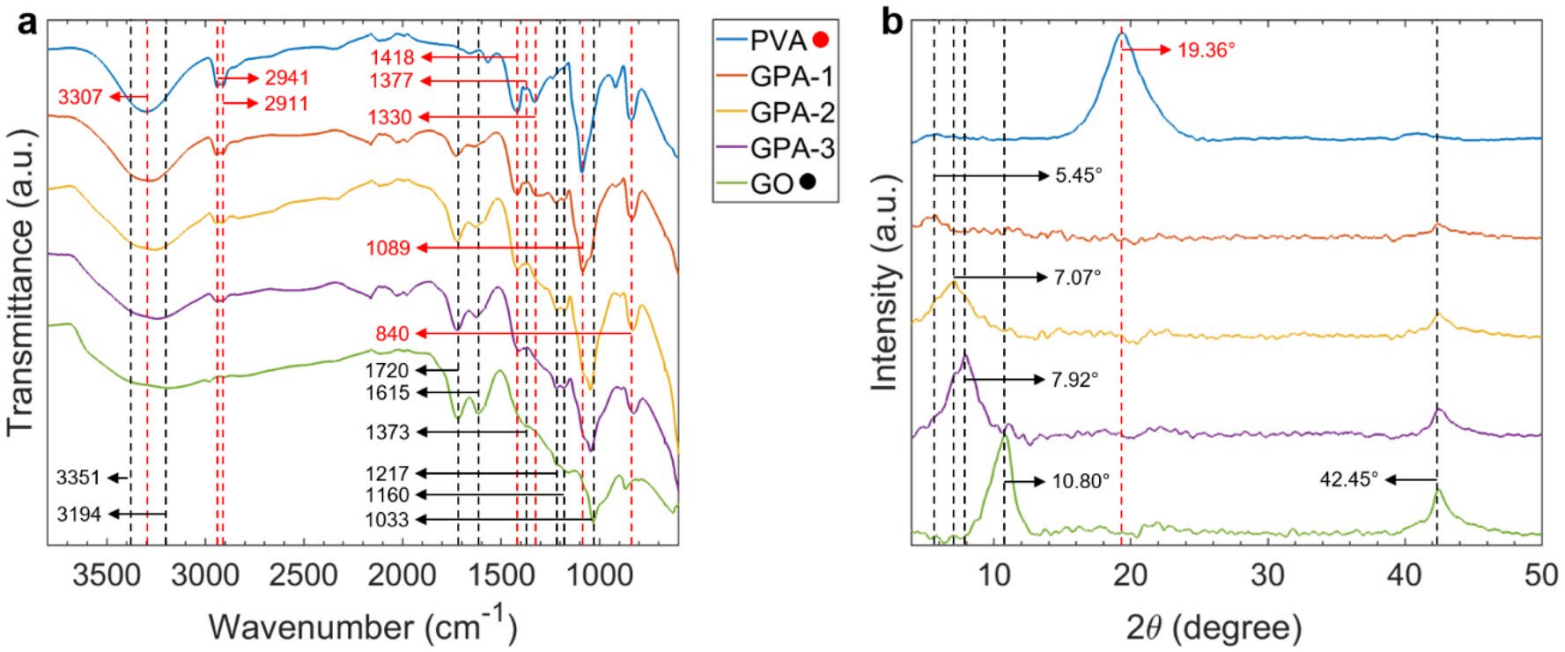
Physicochemical characterisation of GO/PVA aerogels: (**a**) FT-IR spectra and (**b**) XRD patterns. Wavenumbers attributable to GO are denoted in black while those for PVA are in red.

Figure 3a shows the FT-IR spectra for all the samples. The main features observable in the GO spectrum are the O–H stretching and deformation of hydroxyl groups at 3351 and 1373 cm^−1^, coupled with the C–OH stretching at 1217 and 1160 cm^−1^, the C=O stretching of carbonyl groups at 1718 cm^−1^, and the C–O–C stretching of epoxy groups at 1033 cm^−1^^35–38^. The peaks at 3194 and 1615 cm^−1^ are respectively due to the stretching and deformation of adsorbed water molecules^38^. On the other hand, the main features of the PVA spectrum are the O–H stretching and deformation at 3307 and 1377 cm^−1^, the asymmetric and symmetric stretching of C–H at 2941 and 2911 cm^−1^ respectively, the C–H_2_ bending at 1418 cm^−1^, the C–O–C stretching at 1089 cm^−1^, and the C–C stretching at 845 cm^−1^^39–41^. As GO/PVA blends exhibit the features of both the components, with intensities proportional to their relative mass ratios, the homogeneous mixing and stability of the samples can be assumed. Additionally, a shift of O–H related features around 3351, 1373, 1217, and 1160 cm^−1^ confirms the formation of hydrogen bonds between the oxygen groups on GO sheets and the hydroxyl groups of PVA molecules^42–44^.

From XRD patterns presented in Fig. 3b, GO shows its characteristic peak associated with the (001) carbon crystalline phase at 10.80° and the (100) reflection related to the longitudinal dimension of the structural elements at 42.45°^45,46^. The introduction of increasing PVA amounts in the blends affects the (001) peak causing its reduction in intensity and sharpness. This indicates a lower degree of crystallinity and a possible decrease in the size of crystallites. A shift toward lower $2\theta$ values can also be observed, indicating that the interactions between PVA molecules and GO sheets lead to a more expanded structure. According to Bragg’s law^47^, the interplanar distance ($d$) between GO layers increases from 8.19 Å for pristine GO to the maximum of 16.20 Å for GPA-1. The PVA main reflection (101) peak appearing at 19.36° is not visible in the patterns for GPAs, which is the sign of a completely amorphous phase^48^.

### Optimisation of the acoustic properties

**Figure 4:**
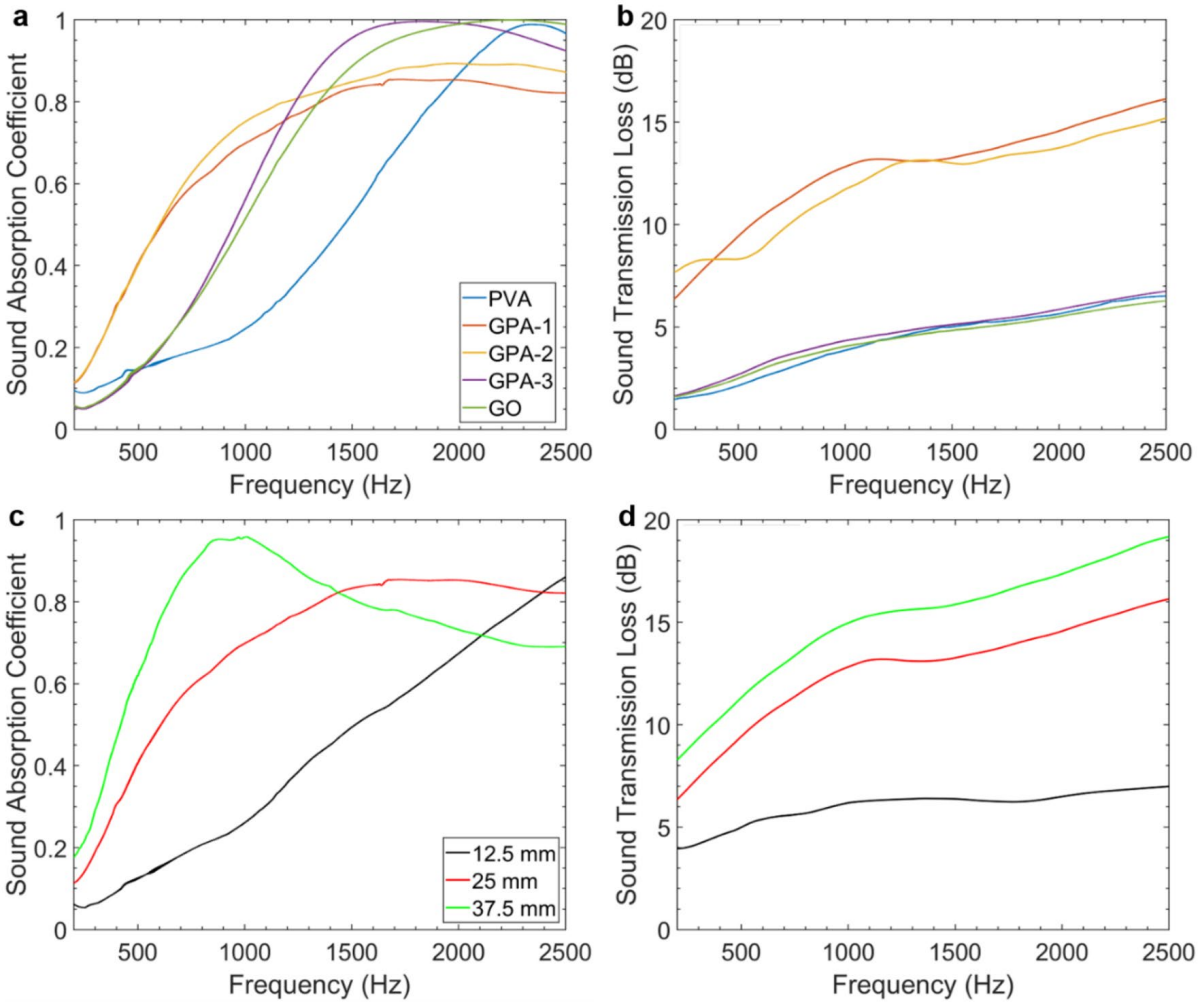
Effects of (**a**,**b**) GPA composition and (**c**,**d**) GPA-1 thickness on acoustic properties: (**a**,**c**) sound absorption and (**b**,**d**) sound transmission loss. Sample thickness is 25 mm when not studied as a variation (**a**,**b**). Key and average $\alpha$ and $STL$ values are summarised in Tables S2 and S3.

Figure 4a,b presents the variation of $\alpha$ and $STL$ for different compositions of GPAs as a function of frequency. As PVA inclusion increases, the absorption curves are flattened and shift toward lower frequencies than pure GO. This leads to higher absorption in the low frequency range (i.e., below 1200 Hz), with $\alpha$ > 0.4 from 500 Hz for GPA-1 and GPA-2. GPA-3 and pure GO outperform in the high frequency range (i.e., above 1200 Hz), and have similar performances to other GO foams reported in the literature^8^. This behaviour is related to changes in the physical structure of the aerogel. In particular, the macrostructure of GPA-1 and GPA-2 exhibits large pores (Fig. 2b,c) that result to an increased porosity and a reduced flow resistivity. The average sound absorption coefficients ($\overline{\alpha }$) for all the GPA samples fall in the 0.74 and 0.77 range. A PVA inclusion higher than 75 wt% (GPA-1 and GPA-2) results in improved transmission loss performances (Fig. 4b), with GPA-1 having the highest average loss ($\overline{STL}$) of 13.2 dB. The $STL$ generally reflects the damping properties attributed to the cross-sectional distribution of large and small pores within the aerogel (Fig. 2g), and therefore sound attenuation through the material^49^. The relation between $\alpha$ and the Reflection Coefficient ($R$), $\alpha = 1 - \left| R \right|^{2}$, further justifies this behaviour as higher transmission losses are expected from a structure showing lower absorption and, consequently, higher reflections.

GPA-1 was chosen as the optimum composition as it possesses high sound absorption and sound transmission losses while being the lightest aerogel. Figure 4c,d shows the effect of adjusting the thickness from 12.5 to 37.5 mm on both $\alpha$ and $STL$.The increase in aerogel thickness improves sound absorption at lower frequencies as expected, with the 37.5 mm thick sample achieving $\alpha$ > 0.6 from 500 Hz upwards with a peak of $\alpha$ = 0.96 at 948 Hz. $\overline{STL}$ follows the same behaviour and reaches 15.7 dB for 37.5 mm thickness.

**Figure 5:**
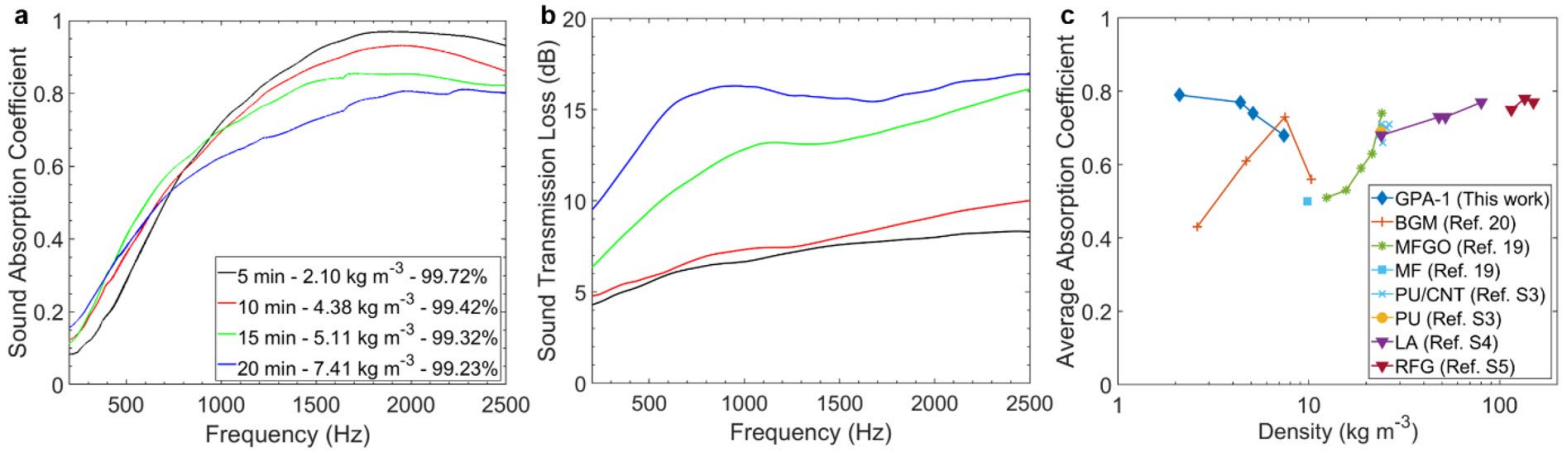
Effects of processing time and densities on acoustic properties of GPA-1 samples: (**a**) sound absorption and (**b**) sound transmission loss. Key and average $\alpha$ and $STL$ values are summarised in Table S4. (**c**) Comparison of the average sound absorption coefficient, calculated in the 400–2500 Hz range, as a function of density for GPA-1 samples and other porous absorbers with comparable thickness previously reported in literature (Table S1).

The next optimisation step is the evaluation of processing time and, consequently, porosity on acoustic properties of GPA-1. Figure 5a shows that an increasing porosity leads to higher sound absorption of the proposed structure over the frequency range investigated. In particular, the lightest aerogel obtained with 5 min of processing time results in a density of 2.10 kg m^−3^ and a porosity of 99.72% achieving $\overline{\alpha }$ = 0.79. As sound waves travel from large to smaller pores (Fig. 2g), air velocity increases and sound energy is dissipated due to friction^50,51^. Figure 5b depicts an increase of the transmission loss as aerogels become bulkier. The heaviest aerogel (7.41 kg m^−3^) has the best result with $\overline{STL}$ = 15.8 dB. Furthermore, Fig. 5c compares $\overline{\alpha }$ and density values of GPA-1 aerogels with other porous absorbers previously reported, demonstrating their superior acoustic properties while guaranteeing extremely low densities.

### Semi-phenomenological analysis

**Figure 6:**
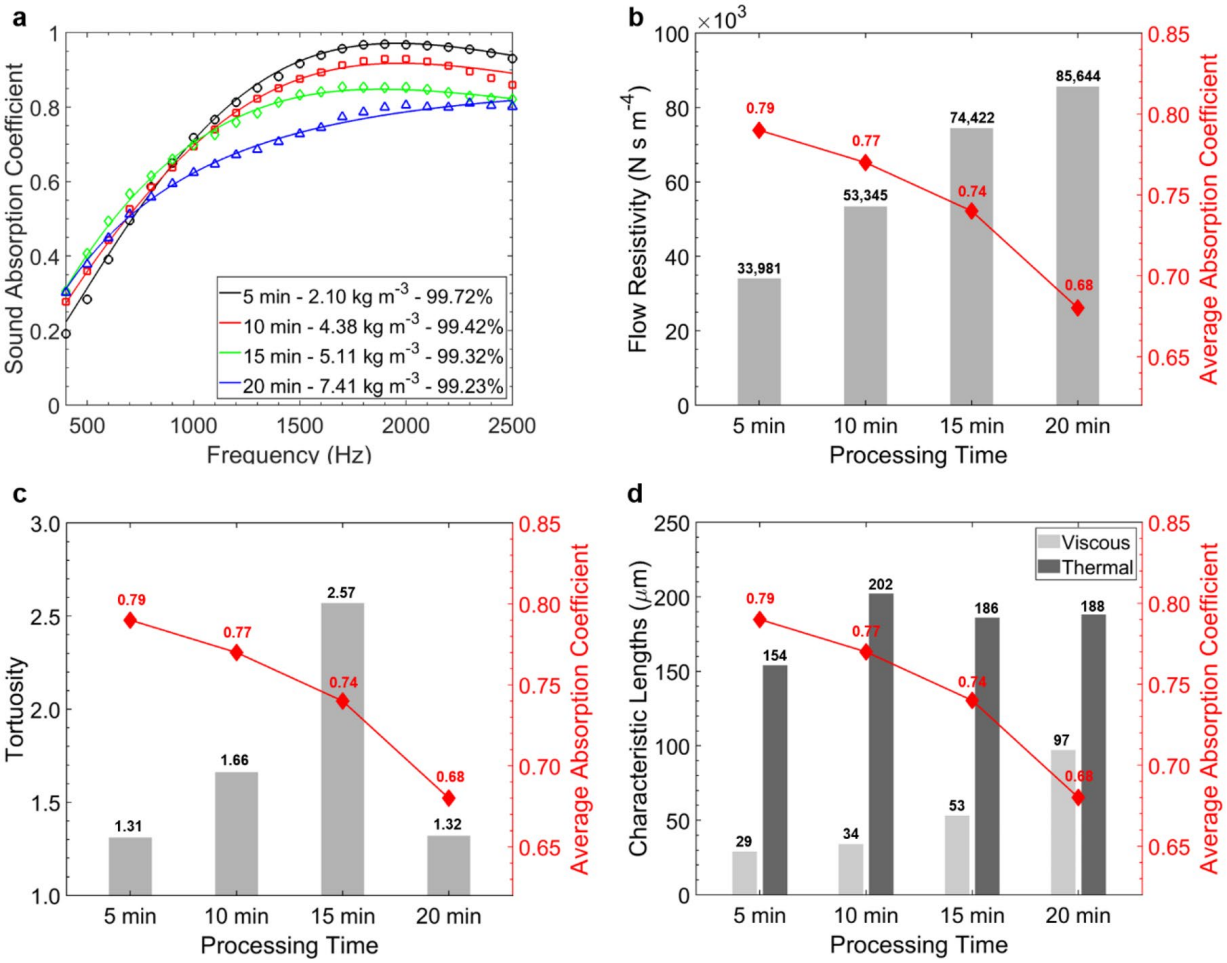
Semi-phenomenological analysis of GPA-1 samples with different processing times: (**a**) comparison between semi-analytical model predictions (solid lines) and experimental measurements (markers) of the sound absorption coefficient. (**b**) Flow resistivity, (**c**) tortuosity, (**d**) viscous and thermal characteristic lengths affected by processing time with average absorption coefficient trend.

The effects of processing time on the physical structure of GPA-1 and the resulting acoustic behaviour are further investigated using the JCA semi-phenomenological approach. The equivalent fluid model is fitted to measured results considering three experimentally derived parameters ($\phi$, $\sigma$ and $\alpha_{\infty }$) and two unknown parameters ($\Lambda$ and $\Lambda ^{\prime}$). The predicted sound absorption coefficient curves have an average error < 1% when compared to the experimental results (Fig. 6a), suggesting a good fit to the non-acoustic properties of the aerogels. For different processing times, the density and the non-acoustic properties change, and the complex interactions between these parameters lead to the observed shift in GPA-1 sound absorption. Porosity is directly proportional to the volume of air available to sound waves^52^ and positively contributes to sound energy dissipation. However, it is crucial to highlight that the non-acoustic properties are not independent (i.e., a change in one parameter will cause a change in the others). An inverse trend of $\overline{\alpha }$ with respect of $\sigma$ can be observed from Fig. 6b. In particular, the best performing sample (5 min of processing time) shows $\overline{\alpha }$ = 0.79 for $\sigma$ = 33,981 N s m^−4^. The flow resistivity is a measure of the porous material’s resistance to an airflow and can thus give an idea of the extent of sound energy dissipation due to boundary layer effects within the material^52^. However, if the resistivity is too high the sound wave incident to the material would meet a relatively high impedance surface leading to high reflection due to the impedance mismatch and thus to low sound absorption^53^. This is in agreement with $STL$ results previously reported (Fig. 5b). The variation in $\alpha_{\infty }$ shown in Fig. 6c is within a relatively small range of 1.31–1.66, except for the 15 min sample. The tortuosity is a measure of the complexity of the propagation path of sound waves through the material, where more complex paths usually lead to higher sound absorption. However, no direct correlation is exhibited between $\overline{\alpha }$ and $\alpha_{\infty }$ for the experimental aerogels. $\Lambda$ increases with processing time from the minimum value of 29 µm to the maximum of 97 µm (Fig. 6d). The viscous characteristic length is defined as “the surface-to-pore volume ratio of the pore-solid interface”^9^ and is thus proportional to the microscopic dimensions of pores. Smaller values lead to increased viscous effects and thus to an improved dissipation of sound energy, explaining the acoustic behaviour pictured in Fig. 6a. Finally, the variation of $\Lambda ^{\prime}$ (between 154 and 202 µm) is a function of thermal losses at high frequencies^52^ and thus has limited effect on sound absorption over the range investigated (Fig. 6d).

## Discussion

In conclusion, we have developed a novel ultralight aerogel for the design of thin and light materials with excellent acoustic properties. We have exploited the chemical properties of GO/PVA blends and a specific environmentally friendly manufacturing process to embed the aerogels in structural HC cores. The physicochemical characterisation has demonstrated the effects of the blend composition on the physical properties of the material, the existence of hydrogen bonds between GO sheets and PVA molecules and the ability of the two components to form a homogeneous and expanded structure. We have also evaluated the effects of composition, thickness, and processing time on the acoustic properties of the proposed material. Thanks to the hierarchical porosity, the resulting absorber is endowed with the advantages of a density as low as 2.10 kg m^−3^, and tuneable sound absorption and transmission functionality. The novel aerogel-based structures provide a solution for the development of acoustic materials in structural engineering applications requiring high sound absorption and sound transmission losses as well as excellent mechanical stiffness and strength. Additionally, the inherent potential of GO to unlock multifunctional features such as EMI shielding and fire-retardancy may prompt advanced applications in the aerospace and power generation industries.

## Methods

### Materials

Graphite oxide (GtO) powder was supplied by Xiamen TOB New Energy, PVA (98–99% hydrolyzed, medium molecular weight) was purchased from Sigma Aldrich. Deionized MilliQ^®^ water was used throughout all the experiments. All the chemicals were used as received without further treatment or purification.

### Sample fabrication

**Figure 7:**
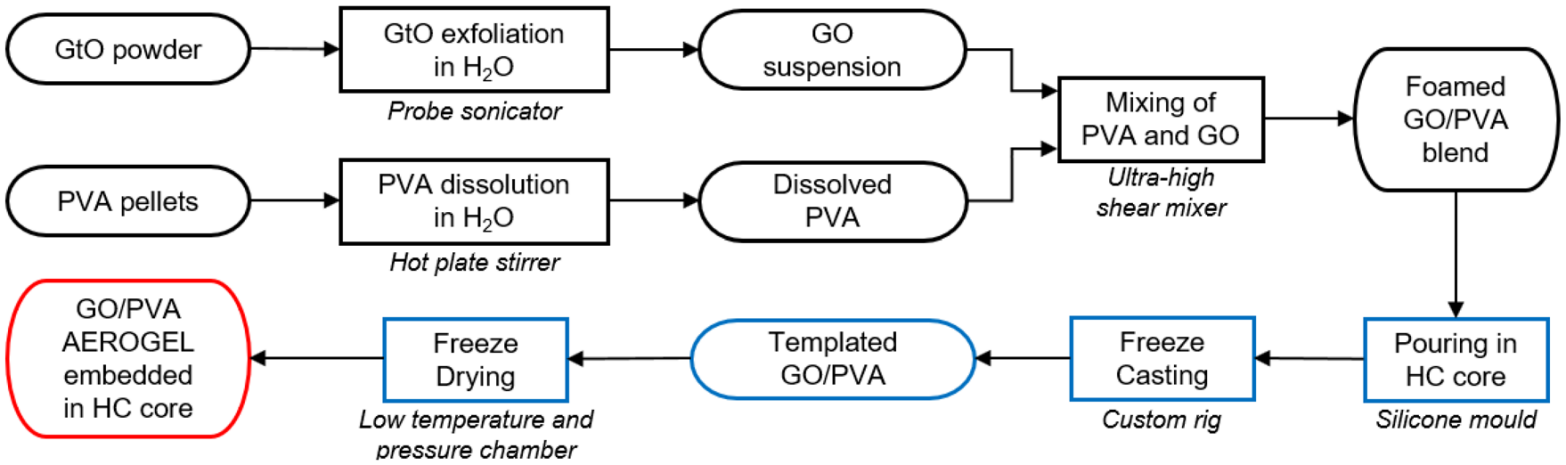
Diagram of ultralight GO/PVA aerogel fabrication process.

The fabrication process is schematised in Fig. 7. GtO was dispersed in water (8 mg mL^−1^) and exfoliated to form a GO suspension through probe sonication (Dr. Hielscher GmbH UP100H, with an amplitude of 80% and continuous pulsing) for 40 min under constant magnetic stirring and in an ice bath to ensure a homogeneous process with controlled temperature. A PVA solution (5 wt%) was obtained by dissolving the raw polymer in water: the system was heated up to 90 °C on a hot plate with continuous magnetic stirring until the solution became clear. Blends of GO and PVA were then obtained with ultra-high shear mixing (IKA Ultra-Turrax T25) at 20,000 rpm for 15 min in a typical blend, or with varying processing times for porosity optimisation purposes. Proper amounts of the two components were mixed so that the designed mass ratio could be reached (Table 1).

Aerogels were obtained with unidirectional freeze-casting of the blends in a Nomex HC core with the aid of a silicone mould having an aluminium plate as base directly placed on a copper heat sink immersed in liquid nitrogen. Templated structures were finally freeze-dried (LTE LyoTrap Mini) for 96 h, leaving the GPAs embedded in the Nomex core. Samples consisting of pure GO suspension and pure PVA solution were also manufactured as reference material.

### Characterisation

The composition of the starting blend determined the morphology of the aerogels, evaluated with DM (Keyence VHX 6000) and SEM (Hitachi SU3900). Chemical structure was evaluated by Fourier-Transform Infrared Spectroscopy (FT-IR, Perkin-Elmer Frontier FTIR Spectrometer) with a liquid nitrogen cooled MCT detector from 400 to 4000 cm^−1^. The crystalline structure of the blends was finally analysed with X-Ray Diffractometry (XRD, STOE STADI P) in the range of $2\theta = 4{-}50^\circ$ at room temperature using a Cu-Kα generator with 1.54 Å wavelength. XRD data were additionally processed to calculate the interplanar distance $d$ between GO layers using Bragg’s law^47^ as expressed in Eq. (1), where $\lambda$ is the radiation wavelength and $\theta$ is the reflection angle of the (001) phase.

1 $$ d = {\raise0.7ex\hbox{$\lambda $} \!\mathord{\left/ {\vphantom {\lambda {2\sin \theta }}}\right.\kern-\nulldelimiterspace} \!\lower0.7ex\hbox{${2\sin \theta }$}} $$

The density of the samples ($\rho_{s}$) was calculated from their weight and volume. The porosity of each sample was calculated as expressed in Eq. (2), where $\rho_{GO}$ and $\rho_{PVA}$ are the densities of bulk GO (0.26 g cm^−3^) and PVA (1.25 g cm^−3^), respectively, while $w_{GO}$ and $w_{PVA}$ are the mass percentages of the two components in the blend.

2 $$ \phi = \left( {1 - \frac{{\rho_{s} }}{{w_{GO} \rho_{GO} + w_{PVA} \rho_{PVA} }}} \right) \times 100 $$

The coefficients expressing acoustic performances, $\alpha$ and $STL$, were measured following the standard test methods ASTM E1050^54^ and ASTM E2611^55^, respectively. Detailed experimental procedures can be found in Supplementary Information.

Detailed information on the measurement of the non-acoustic properties can be found in Supplementary Information. Briefly, porosity was evaluated using the density of the aerogels as expressed in Eq. (2), flow resistivity was indirectly determined from impedance tube measurements according to equation (S9)^55–57^, tortuosity was experimentally derived from equation (S10) using an ultrasonic time-of-flight method^58^, and finally viscous and thermal characteristic lengths were obtained by applying an inverse identification method^59,60^.

## Supplementary Information

Supplementary Information.
