# Supplementary material for: Ultralight graphene oxide/polyvinyl alcohol aerogel for broadband and tuneable acoustic properties
Source: Sci Rep. 2021 May 19;11:10572. doi: 10.1038/s41598-021-90101-0 (PMC8134629; doi:10.1038/s41598-021-90101-0)
Supplement: Supplementary file 1 — Supplementary Information. [file 41598_2021_90101_MOESM1_ESM.pdf]

Ultralight Graphene Oxide/Polyvinyl Alcohol Aerogel for broadband and tuneable acoustic properties

SUPPLEMENTARY INFORMATION

Mario Rapisarda[1], Gian-Piero Malfense Fierro[1], and Michele Meo[1,*]

[1]*University of Bath, Department of Mechanical Engineering, Bath, BA27AY, UK*
[*]*m.meo@bath.ac.uk*

Table S1. Comparison of density, porosity, and sound absorption properties between GPA-1 samples from this work and other porous absorbers with comparable thickness previously reported in the literature.
[A] The average was calculated in the 400 – 2500 Hz range.

Name	Density (kg m^{-3})	Porosity (%)	Thickness (mm)	Average[A] Absorption Coefficient	Ref.
GPA-1	2.10 – 7.80	98.61 – 99.72	25	0.68 – 0.79	This work
BGM	2.6 – 10.3	up to 99.7	30	0.43 – 0.73	1
MFGO	12.39 – 24.12	-	26	0.51 – 0.74	2
Commercial MF	9.84	-	26	0.50	2
PU/CNT foams	23.37 – 26.31	~95	30	0.66 – 0.71	3
Commercial PU	23.87	~95	30	0.69	3
Lignin Aerogels	24 – 80	94 – 98.4	-	0.68 – 0.77	4
RFG	115 – 150	94 – 95.4	30	0.75 – 0.77	5

Table S2. Sound absorption and transmission properties for GPAs with different composition.
[A] The average was calculated in the 400 – 2500 Hz range.

Sample	Peak		500 Hz		1000 Hz		2000 Hz		Average[A]	
	Freq (Hz)	α	α	STL (dB)	α	STL (dB)	α	STL (dB)	$\overline{\alpha}$	$\overline{STL}$ (dB)
PVA	2339	0.99	0.15	2.1	0.25	3.9	0.87	5.65	0.54	4.6
GPA-1	1734	0.85	0.41	9.4	0.70	12.8	0.85	14.57	0.74	13.2
GPA-2	1970	0.89	0.41	8.3	0.75	11.7	0.89	13.75	0.77	12.4
GPA-3	1827	1.00	0.15	2.7	0.56	4.3	0.99	5.86	0.74	4.9
GO	2244	1.00	0.15	2.5	0.51	4.1	0.99	5.51	0.72	4.6

Table S3. Sound absorption and transmission properties for GPA-1 samples with different thicknesses.
[A] The average was calculated in the 400 – 2500 Hz range.

Sample	Peak		500 Hz		1000 Hz		2000 Hz		Average[A]	
	Freq (Hz)	α	α	STL (dB)	α	STL (dB)	α	STL (dB)	$\overline{\alpha}$	$\overline{STL}$ (dB)
12.5 mm	2500	0.86	0.13	5.0	0.26	6.2	0.67	6.49	0.46	6.2
25 mm	1735	0.85	0.41	9.4	0.70	12.8	0.85	14.57	0.74	13.2
37.5 mm	948	0.96	0.62	11.3	0.96	15.0	0.73	17.36	0.79	15.7

Table S4. Sound absorption and transmission properties for GPA-1 samples obtained with different mixing times.
[A] The average was calculated in the 400 – 2500 Hz range.

Sample	Peak		500 Hz		1000 Hz		2000 Hz		Average[A]	
	Freq (Hz)	α	α	STL (dB)	α	STL (dB)	α	STL (dB)	$\overline{\alpha}$	$\overline{STL}$ (dB)
5 min	1885	0.97	0.29	5.5	0.72	6.7	0.97	8.00	0.79	7.3
10 min	1952	0.93	0.36	5.8	0.69	7.3	0.93	9.13	0.77	8.0
15 min	1735	0.85	0.41	9.4	0.70	12.8	0.85	14.57	0.74	13.2
20 min	2284	0.81	0.38	13.7	0.62	16.3	0.80	16.11	0.68	15.8

Methods used to measure acoustic properties.

The aerogels were acoustically characterised through measurements of two key parameters: the Normal Absorption Coefficient (α) and the Normal Incident Sound Transmission Loss (STL). For the first, the standard test method ASTM E1050[6] was followed. Briefly, samples of the composite structure were placed in one end of a two microphone impedance tube having an internal diameter of 50.8 mm with a rigid back surface, while a loudspeaker generating a broadband random signal was mounted at the other end. The coefficient α was then estimated as expressed in equation (S1):

$$\alpha = 1 - |R|^2 \tag{S1}$$

Where R is the Complex Reflection Coefficient measured on the incident surface of the sample following the transfer function method[6].

The sound transmission losses were instead evaluated according to the standard test method ASTM E2611[7]. The procedure is similar to the determination of α, but the transfer functions were calculated for four microphones, with two of them mounted each side of the sample, and two different terminations, anechoic and open. The STL was then estimated with equation (S2):

$$STL = 10\log_{10}\left(\frac{1}{\tau}\right) \tag{S2}$$

where τ is the sound transmission coefficient.

Equivalent fluid model of porous absorbers.

The acoustic behaviour of GPA-1 samples, specifically in terms of sound absorption ability, was studied with a semi-phenomenological approach following the Johnson-Champoux-Allard (JCA) model for porous materials[8,9]. The effective density (ρ_e) and effective bulk modulus (K_e) relate the physical properties of the absorber to the sound propagation through it, and are calculated as expressed in equations (S3) and (S4):

$$\rho_e = \frac{\alpha_\infty \rho_0}{\phi}\left(1 + \frac{\sigma\phi}{i\omega\rho_0\alpha_\infty}\sqrt{1 + \frac{4i\alpha_\infty{}^2\eta\rho_0\omega}{\sigma^2\Lambda^2\phi^2}}\right) \tag{S3}$$

$$K_e = \frac{\gamma P_0}{\phi}\left[\gamma - (\gamma - 1)\Big/\left(1 + \frac{8\eta}{i\Lambda'^2 N_{pr}\omega\rho_0}\sqrt{1 + \frac{i\rho_0\omega N_{pr}\Lambda'^2}{16\eta}}\right)\right]^{-1} \tag{S4}$$

where ρ_0, η, γ, and N_{pr} are density, dynamic viscosity, ratio of the specific heat capacities and Prandtl Number for air, respectively, while P_0 is the atmospheric pressure. The remaining parameters (i.e., the non-acoustic properties of porous materials) are porosity (ϕ), flow resistivity (σ), tortuosity (α_∞), viscous (Λ) and thermal (Λ') characteristic lengths.

The characteristic impedance (Z_c) and wavenumber (k_c) can then be determined from equation (S5) and (S6), respectively:

$$Z_c = \sqrt{K_e \rho_e} \tag{S5}$$

$$k_c = \omega \sqrt{\frac{\rho_e}{K_e}} \tag{S6}$$

These equations allow the calculation of the normal incidence surface impedance (Z_s), R and α, as expressed in equations (S7), (S8) and (S2), respectively.

$$Z_S = -i\, Z_c \cot(k_c d) \tag{S7}$$

$$R = \frac{Z_S - Z_0}{Z_S + Z_0} \tag{S8}$$

Methods to measure the non-acoustic properties.

The porosity was calculated as expressed by equation (2) in the manuscript.

The flow resistivity was indirectly measured from the low frequency acoustic behaviour of the samples in a standard impedance tube with two different terminations (i.e., anechoic and open), according to equation (S9)[10,11]:

$$\sigma = \lim_{\omega \to 0} [-Im(Z_c k_c)] \tag{S9}$$

where Z_c and k_c were evaluated following the transfer matrix approach detailed in the standard test method ASTM E2611[7].

The tortuosity was determined with ultrasonic wave speed measurements in a sample saturated by air, according to equation (S10)[12]:

$$\alpha_\infty = \left(\frac{C_0}{C}\right)^2 (1 - 2\varphi) \tag{S10}$$

where the ratio of celerity in free air over velocity inside the porous material (C_0/C) was calculated from the increase of the time of flight of a short ultrasonic pulse sent at 50 kHz between two transducers when a sample of the material was inserted. The loss angle (φ) was derived from the pulse signal damping.

The viscous and thermal characteristic lengths were finally obtained using an inverse identification method[13,14]: The sound absorption coefficient predicted by the JCA model was fitted to the acoustical experimental data by varying Λ and Λ' and keeping all the other non-acoustic properties fixed to the experimentally or indirectly derived values.

References

1. Lu, B. *et al.* High performance broadband acoustic absorption and sound sensing of a bubbled graphene monolith. *Journal of Materials Chemistry A* **7**, 11423-11429, doi:10.1039/C9TA02306B (2019).

2. Nine, M. J. *et al.* Graphene Oxide-Based Lamella Network for Enhanced Sound Absorption. *Advanced Functional Materials* **27**, 1703820, doi:https://doi.org/10.1002/adfm.201703820 (2017).

3. Hasani Baferani, A., Katbab, A. A. & Ohadi, A. R. The role of sonication time upon acoustic wave absorption efficiency, microstructure, and viscoelastic behavior of flexible polyurethane/CNT nanocomposite foam. *European Polymer Journal* **90**, 383-391, doi:https://doi.org/10.1016/j.eurpolymj.2017.03.042 (2017).

4. Wang, C. *et al.* Cellulose as an adhesion agent for the synthesis of lignin aerogel with strong mechanical performance, Sound-absorption and thermal Insulation. *Scientific Reports* **6**, 32383, doi:10.1038/srep32383 (2016).

5. Sun, Z., Shen, Z., Ma, S. & Zhang, X. Sound absorption application of fiberglass recycled from waste printed circuit boards. *Materials and Structures* **48**, 387-392, doi:10.1617/s11527-013-0190-6 (2015).

6. ASTM E1050-19, Standard Test Method for Impedance and Absorption of Acoustical Materials Using a Tube, Two Microphones and a Digital Frequency Analysis System, ASTM International, West Conshohocken, PA, 2019,. doi:10.1520/E1050-19.

7. ASTM E2611-19, Standard Test Method for Normal Incidence Determination of Porous Material Acoustical Properties Based on the Transfer Matrix Method, ASTM International, West Conshohocken, PA, 2019,.

8. Johnson, D. L., Koplik, J. & Dashen, R. Theory of dynamic permeability and tortuosity in fluid-saturated porous media. *Journal of Fluid Mechanics* **176**, 379-402, doi:10.1017/S0022112087000727 (1987).

9. Champoux, Y. & Allard, J. F. Dynamic tortuosity and bulk modulus in air - saturated porous media. *Journal of Applied Physics* **70**, 1975-1979, doi:10.1063/1.349482 (1991).

10. Tao, J., Wang, P., Qiu, X. & Pan, J. Static flow resistivity measurements based on the ISO 10534.2 standard impedance tube. *Building and Environment* **94**, 853-858, doi:https://doi.org/10.1016/j.buildenv.2015.06.001 (2015).

11. Doutres, O., Salissou, Y., Atalla, N. & Panneton, R. Evaluation of the acoustic and non-acoustic properties of sound absorbing materials using a three-microphone impedance tube. *Applied Acoustics* **71**, 506-509, doi:https://doi.org/10.1016/j.apacoust.2010.01.007 (2010).

12. Allard, J. F., Castagnede, B., Henry, M. & Lauriks, W. Evaluation of tortuosity in acoustic porous materials saturated by air. *Review of Scientific Instruments* **65**, 754-755, doi:10.1063/1.1145097 (1994).

13. Atalla, Y. & Panneton, R. Inverse acoustical characterization of open cell porous media using impedance tube measurements. *Canadian Acoustics* **33**, 11-24 (2005).

14. Fellah, Z. E. A., Mitri, F. G., Fellah, M., Ogam, E. & Depollier, C. Ultrasonic characterization of porous absorbing materials: Inverse problem. *Journal of Sound and Vibration* **302**, 746-759, doi:https://doi.org/10.1016/j.jsv.2006.12.007 (2007).
